# P-1917. Targeted Needs Assessment for a Web-Based Infectious Disease Learning Tool: Applying Kern’s Six-Step Approach

**DOI:** 10.1093/ofid/ofaf695.2086

**Published:** 2026-01-11

**Authors:** Thuy Anh Vo, Colette Bare, Sabrina Shih, Melody Chiang, Emily Abdoler

**Affiliations:** University of Michigan Medical School, Silver Spring, MD; University of Michigan Medical School, Silver Spring, MD; University of Michigan, Ann Arbor, Michigan; University of Michigan, Ann Arbor, Michigan; University of Michigan, Ann Arbor, Michigan

## Abstract

**Background:**

As medicine advances and preclinical learning phases are shortened, medical students face an increasingly information-dense curriculum. At the University of Michigan Medical School (UMMS) – where the preclinical curriculum is largely lecture-based and asynchronous over a 12 month period– local web-based resources have enhanced student engagement and knowledge retention in several content areas. However, no such learning resource exists for preclinical Infectious Disease (ID)/Microbiology. To fill this gap, we used Kern’s approach to curriculum development as part of the tool design process, starting with a targeted needs assessment.

Medical Students' Preferred Topics

**Figure 1:**
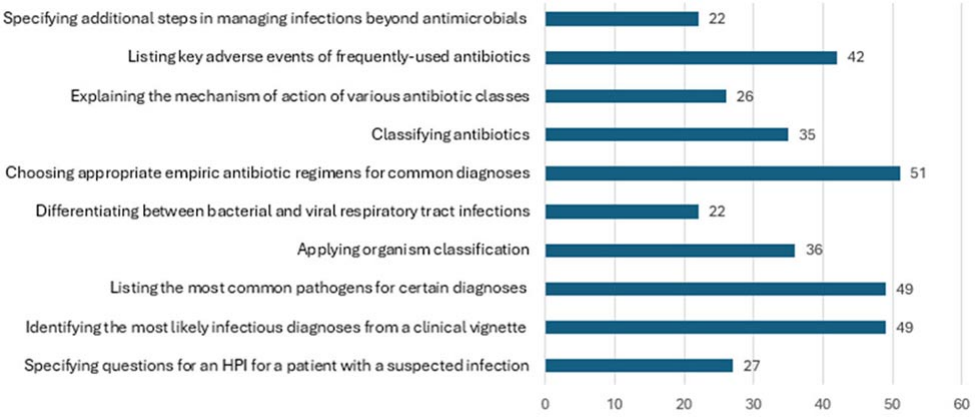
Specific components students would included in a web-based ID/Microbiology co-curricular supplemental module.

**Methods:**

We designed an anonymous survey with Likert-scale items to assess student satisfaction with, preparedness for clinical application of, and interest in supplemental resources for the ID/Microbiology content. Students were also asked to choose preferred features for an online learning tool.

**Results:**

Fifty-three medical students in the 2nd through 4th years responded (10% response rate). Only 62% of students reported being at least partially satisfied with opportunities to apply their ID knowledge in the pre-clinical curriculum. In terms of clinical readiness, 55% felt at least somewhat prepared to diagnose infections, while only 17% felt at least somewhat prepared to propose initial treatment plans for infections. Notably, 94% of respondents expressed interest in a web-based ID supplement, with their feedback informing specific components for inclusion in the proposed tool (Figure 1).

**Conclusion:**

This needs assessment highlights significant gapsin students’ preparation to apply ID/Microbiology knowledge in a clinical context and affirms student interest in an interactive web-based tool to address this need. We have used Kern’s steps to design MicroModules, asynchronous case-based modules that emphasize clinical reasoning and the application of microbiology in a clinical context. Following implementationthis year, formative and summative evaluations will be used to refine the tool and assess its impact on learner self-efficacy and academic performance. This approach ensures MicroModules is learner-centered and aligned with evolving educational needs.

**Disclosures:**

All Authors: No reported disclosures

